# Inter-relationships of galectin-3 and NLR family pyrin domain containing 3 inflammasomes with oral lichen planus: a preliminary cross-sectional in vitro study

**DOI:** 10.1186/s12903-023-03780-8

**Published:** 2024-01-03

**Authors:** Siting Chen, Xiaoheng Xu, Yang Liu, Yanmei Yao, Yinshen Yang, Wenxia Meng

**Affiliations:** Departments of Oral Medicine, Stomatological Hospital, Southern Medical University NO.366, Jiangnan Road, Guangzhou, Guangdong province 510280 People’s Republic of China

**Keywords:** Galectin-3, NLR family pyrin domain containing 3, Oral lichen planus, Pro-inflammatory state

## Abstract

**Background:**

The nucleotide-binding oligomerization domain (NOD)-like receptor (NLR) family pyrin domain-containing 3 (NLRP3) inflammasome has been reported to be highly expressed in oral lesions with the potential for malignant development such as oral lichen planus (OLP). And the NLRP3 inflammasome can be activated by galectin-3 (Gal-3) in immune-mediated chronic inflammatory diseases. This study aimed to explore the inter-relationships among Gal-3, NLRP3 inflammasome, and OLP.

**Methods:**

A cross-sectional analysis of oral biopsy specimens from 30 patients with Erosive OLP and 30 healthy controls was performed. Immunohistochemical staining was used to evaluate the expression of Gal-3 and NLRP3 inflammasome. Two-sample t-test and Pearson correlation test were applied to analyze the data.

**Results:**

Erosive OLP patients had significantly higher Gal-3 levels compared with controls (*p* < 0.0001). A similar pattern emerged for NLRP3 inflammasome. In the overall sample, a positive correlation was observed between Gal-3 and NLRP3 (*r* = 0.92, *p* < 0.01).

**Conclusions:**

Patients with Erosive OLP lesions showed increased protein expression levels of Gal-3. A positive correlation was observed between Gal-3 and NLRP3 inflammasome.

**Supplementary Information:**

The online version contains supplementary material available at 10.1186/s12903-023-03780-8.

## Introduction

Oral lichen planus (OLP) stands as an immune-mediated chronic inflammatory disease, manifesting through reticulated, linear, and dendritic white markings formed by small papules, often accompanied by congestion, erosion, ulcers, atrophy, and blister formation [1]. Clinically, OLP commonly manifests in a predominantly bilateral and symmetrical distribution. In addition to frequently involving the buccal mucosa, tongue, and gingiva, OLP also affects other mucosal surfaces, particularly the genital region (20%) or the skin (15%) [2–4]. Persistent and recurrent erosion of OLP holds the potential for further malignant transformation, classifying it as an oral potentially malignant disorder (OPMD). The estimated percentage of OLP cases progressing to oral squamous cell carcinoma (OSCC) is between 0.44 and 2.28% [5]. The nucleotide-binding oligomerization domain (NOD)-like receptor (NLR) family pyrin domain-containing 3 (NLRP3) inflammasome is a multimeric complex of proteins comprising NLRP3, apoptosis-associated speck-like protein containing a CARD (ASC), and Caspase-1. When activated, it initiates an inflammatory process [6]. Abnormal activation of NLRP3 inflammasomes can exacerbate inflammation-driven diseases, such as Cryopyrin-associated cyclical syndromes, Alzheimer’s disease, Parkinson’s disease, rheumatoid arthritis, and cancer. Simultaneously, the inflammasome has become a therapeutic target for many diseases [7–9]. Previous reports have indicated increased levels of NLRP3 inflammasome in OLP tissues [10]. Galectin-3 (Gal-3), a unique chimera-type galectin member, exerts diverse immunoregulatory effects in T-cell-mediated inflammatory processes, autoimmune diseases, and tumor progression [11, 12]. In experimental models of autoimmune and chronic inflammatory diseases, including rheumatoid arthritis and atherosclerosis, Gal-3 has been shown to act principally as a pro-inflammatory molecule [13]. Patients with OLP have higher serum levels of Gal-3 compared to healthy controls [14]. However, studies on the expression of Gal-3 in OLP tissues are scarce [15]. Furthermore, despite recent clinical research demonstrating inter-relationships of Gal-3 and NLRP3 inflammasome with primary biliary cholangitis (PBC) and inflammatory bowel disease [16, 17], no studies to date have investigated such inter-relationships in OLP.

To fill this research gap, we examined whether the expression of Gal-3 in OLP patients is higher than that in healthy controls. We also explored potential inter-relationships among Gal-3, NLRP3 inflammasome, and OLP.

## Materials and methods

### Study design and ethical approval

In this cross-sectional analysis, immunohistochemical staining of oral biopsy specimens was performed to evaluate the expression of Gal-3 and NLRP3 inflammasome in patients with OLP and controls. This study was approved by the Institutional Ethics Committee of Stomatological Hospital of Southern Medical University (approval no. (2019)26). All patients provided written informed consent. This study follows the Strengthening the Reporting of Observational Studies in Epidemiology (STROBE) guidelines for reporting cross-sectional studies.

### Tissue samples

Oral biopsy specimens were collected from 30 patients with Erosive OLP and 30 healthy controls. The sample size was determined based on a previous study [10]. Participants were randomly selected from Stomatological Hospital, Southern Medical University, Guangzhou, China, in 2019. In the control group, the tissue samples were obtained from the oral mucosa that needed to be removed during orthognathic operation and were clinically and histopathologically diagnosed as normal oral mucosa. All the healthy controls had no systemic disorders or any other oral lesions. OLP Patients were diagnosed according to clinical and histopathological criteria [18]. Patients with any other oral mucosal diseases, a history of other types of immune-mediated chronic inflammatory diseases, or receiving systemic steroids and immunosuppressive drugs in the previous 4 weeks were excluded. Smokers and alcoholics were excluded from this study. Due to difficulties in obtaining biopsies from age-matching healthy individuals, biopsies from different age range individuals were accepted for inclusion in the control group.

### Immunohistochemistry

All fresh tissue samples were fixed in 4% formalin buffer, embedded in paraffin, sectioned, and then deparaffinized. Antigen retrieval was performed using the wet autoclaving method in the presence of citrate buffer (pH 6.0). After blocking, specimens were incubated overnight at 4 with the following primary antibodies: NLRP3, ASC, Caspase-1 (ABclonal, USA; 1:700, 1:80, 1:30), and Gal-3 (Cell Signalling Technology, USA; 1:200). Next, the sections were incubated with anti-rabbit/mouse-HRP (ZSGB-BIO, China) and 10% normal goat serum at room temperature for 30 min. The DAB REAL EnVision Detection System (Agilent Technologies, USA) was used for color development. The slides were counterstained with modified Harris hematoxylin (Thermo Fisher Scientific, USA). The excess dye solution was washed away and the slides were then mounted using neutral gum mounting film. The sections incubated with phosphate-buffered saline solution were used as negative controls. Images were captured using a digital camera mounted on a light microscope (Leica DM2500 LED). The positive areas of NLRP3, ASC, Caspase-1, and Gal-3 were analyzed using ImageJ software (National Institutes of Health, Bethesda, MD). We used the IHC profiler plug-in to automatically score the stained samples [19]. All assessments were performed in a blinded manner.

### Statistical analysis

Statistical analyses were performed using SPSS Statistics version 17.0 (IBM, USA). After confirming the distribution of the data, two-sample t-tests were used to compare the immunostaining results of Gal-3, NLRP3, ASC, and Caspase-1 between OLP patients and healthy controls. Using the percentages of positive stained areas as continuous variables, the relationship between the NLRP3 inflammasome and Gal-3 in the combined sample was assessed using the Pearson correlation coefficient. A two-tailed *p*-value less than 0.05 was considered to be statistically significant.

## Results

### Participant characteristics

Of the total 60 participants in this study, 35 were females and 25 were males, ranging in age from 19 to 67 (mean age 39.37 ± 13.07 years). The OLP group (*n* = 30) consisted of 18 females and 12 males with an age range of 30–67 years (mean age 48.73 ± 10.66 years). The healthy control group (*n* = 30) comprised 17 females and 13 males with an age range of 19–47 years (mean age 30.00 ± 7.23 years). Overall, 58.33% of study participants were female. Detailed participant data are presented in Table 1.

**Table 1:** Clinical features of the study participants

Groups	Number	Gender		Age (years)	
		Male	Female	Range	Mean ± SD
OLP^a^	30	12	18	30–67	48.73 ± 10.66
NOM^b^	30	13	17	19–47	30.00 ± 7.23
Total	60	25	35	19–67	39.37 ± 13.07

^a^Oral lichen planus; ^b^Normal oral mucosa

### Expression of Gal-3 in oral mucosa of OLP patients and controls

**Fig. 1:**
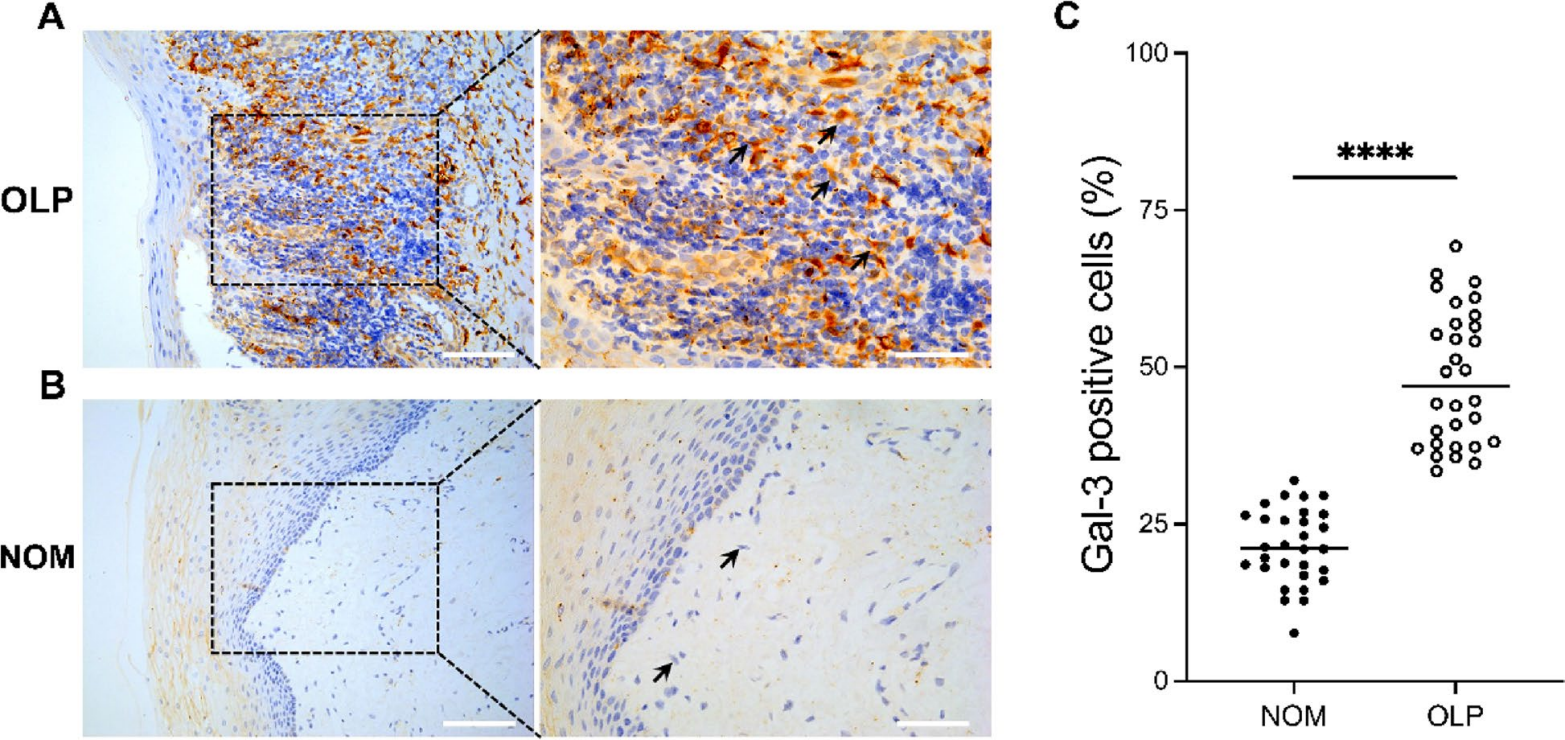
Expression of galectin-3 (Gal-3) in oral lichen planus (OLP) lesions (**A**) and healthy oral mucosa (**B**). Scale bars: 100 μm (left); 50 μm (right). The percentage of Gal-3 positive cells in tissues of OLP patients was significantly higher than that in the control group, t-test; *p* < 0.0001 (**C**)

Immunohistochemistry (IHC) analyses revealed minimal detection of Gal-3 expression in normal stromal cells (Fig. 1A). Conversely, OLP tissues exhibited a noteworthy, moderate-to-intense (yellow-brown/dark brown) homogeneous cytoplasmic expression of Gal-3 (Fig. 1B). The mean Gal-3 expression in OLP tissues (48.32% ± 10.75%) significantly surpassed that in healthy tissues (21.49% ± 5.99%; *p* < 0.0001; Fig. 1C). Gal-3 was predominantly localized in the cytoplasm of cells within the lamina propria beneath the basement membrane. Notably, the cytoplasm in OLP tissues exhibited robust staining, whereas virtually no cytoplasmic staining was observed in normal tissues.

### Expression of NLRP3 inflammasome in OLP patients and controls

**Fig. 2:**
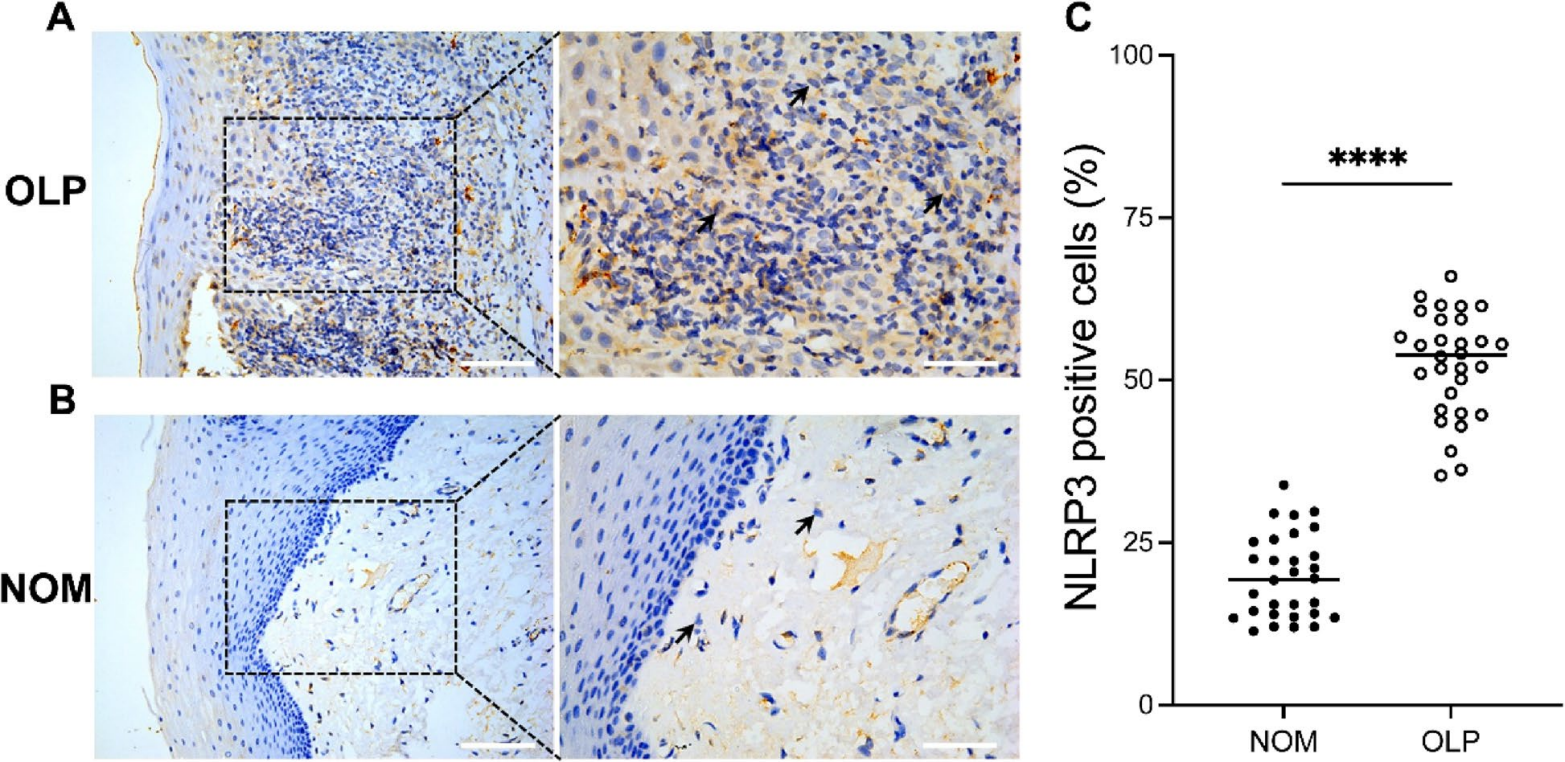
Expression of NLR family pyrin domain containing 3 (NLRP3) in oral lichen planus (OLP) lesions (**A**) and normal oral mucosa (**B**). Scale bars: 100 μm (left); 50 μm (right). The percentage of NLRP3 positive cells in tissues of OLP patients was significantly higher than that in the control group; t-test, *p* < 0.0001 (**C**)

Expression of NLRP3 inflammasome was significantly increased in the lamina propria beneath the basement membrane in tissues from OLP patients (52.42% ± 8.02%) compared to healthy controls (19.74% ± 6.44%; *p* < 0.0001; Fig. 2). Expression of ASC and Caspase-1 was also increased significantly in OLP patients (*p* < 0.001). Further details of ASC and Caspase-1 expression are provided in the Supplementary Material (Fig. S1-S2).

### Correlation among expression of Gal-3 and NLRP3 inflammasome

**Fig. 3:**
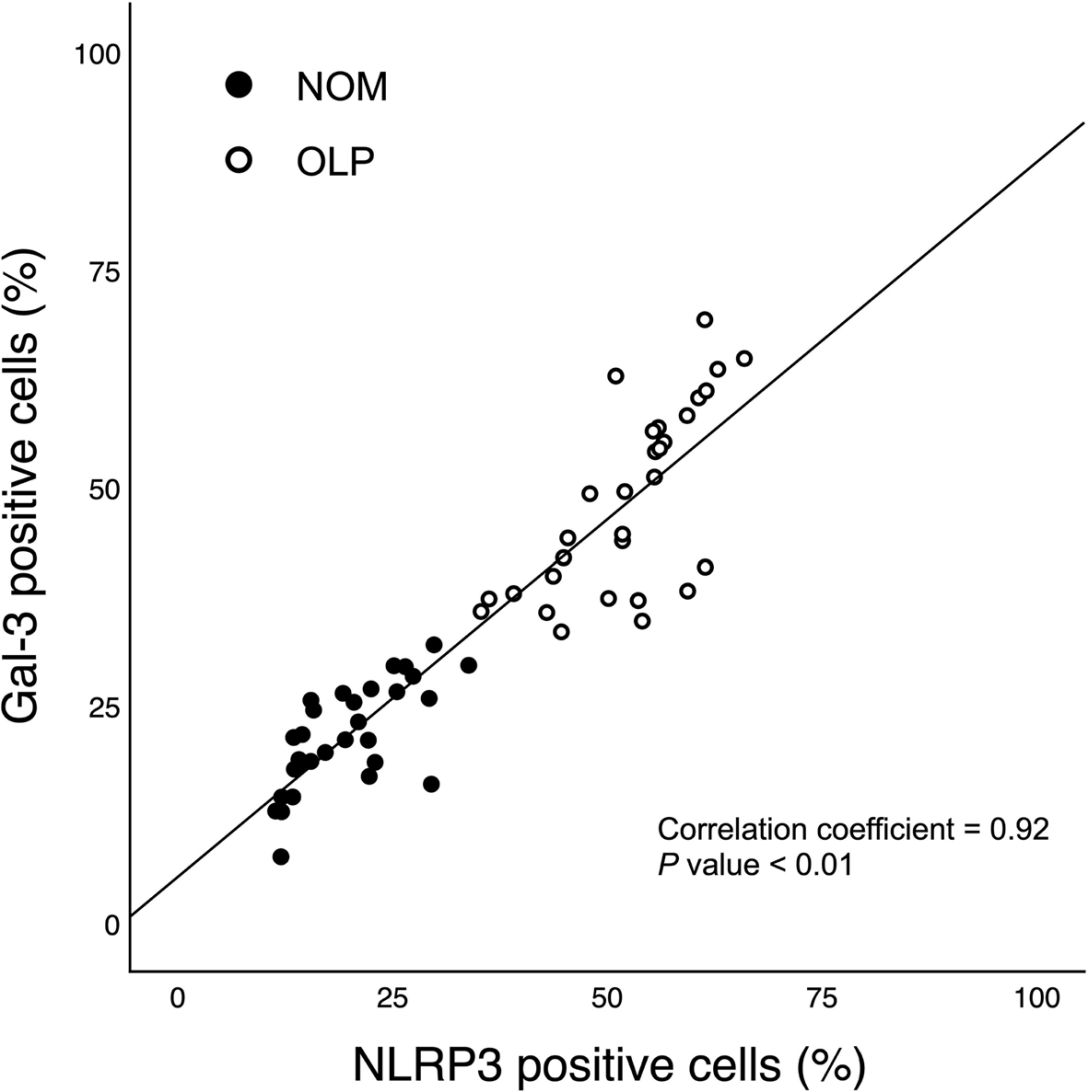
Correlation between the expression of galectin-3 (Gal-3) and NLR family pyrin domain containing 3 (NLRP3) in the overall sample; *r* = 0.92, *p* < 0.01

In the aggregate participant cohort, all correlations between Gal-3 and the NLRP3 inflammasome components (NLRP3, ASC, and Caspase-1) were positively significant (*p* < 0.01). The correlation coefficient between Gal-3 and NLRP3 was 0.92 (Fig. 3), surpassing the coefficients for Gal-3 with the other two constituents of the NLRP3 inflammasome. Supplementary Table S1 furnishes additional details on the Pearson correlation analyses conducted among these molecular entities.

## Discussion

We investigated the expression of Gal-3 and the NLRP3 inflammasome in patients with OLP and healthy controls through immunohistochemistry. Tissue samples obtained from individuals with Erosive OLP exhibited elevated levels of both Gal-3 and the NLRP3 inflammasome when compared to normal oral tissues. Moreover, a noteworthy positive correlation between Gal-3 and NLRP3 expression was observed within the entire sample.

OLP represents a T-cell-mediated chronic inflammatory disorder affecting the oral mucosa [20]. The precise etiological mechanism underlying OLP remains elusive. Multiple factors, including autoimmunity, infection, genetics, psychiatric influences, and other autoimmune diseases, may contribute to OLP. Pathological examination of OLP subepithelial lymphocyte infiltration suggests an inflammatory response. Within OLP, an observed imbalance of helper T cells (Th)1/Th2 and the presence of inflammatory cytokines such as IL-1 and IL-6 have been reported [21, 22]. NLRP3, a crucial protein responsible for pathogen recognition within the body, can be activated by adenosine 5′-triphosphate (ATP), viruses, and sodium urate. This activation promotes the binding of the C-terminal LRR with its ligand, thereby facilitating the formation of the NLRP3 inflammasome complex involving ASC and Caspase-1, ultimately leading to Caspase-1 [23]. The defensive role of inflammasomes in response to pathogens and other danger signals underscores the potential dysregulation of inflammasomes in inflammation-mediated human diseases [24, 25]. In rheumatoid arthritis (RA), the biomarker pentraxin 3, along with its ligand C1q, has been reported to activate NLRP3. Pharmacological inhibition of the NLRP3 inflammasome using various inhibitors has shown reductions in RA pathology and secretion of IL-1β and TNF- α, supporting the implication of NLRP3 in RA [26, 27]. Reports also suggest that NLRP3 inflammasome inhibition might be one mechanism through which metformin mitigates the effects of diabetes on accelerating atherosclerosis [28]. Components of inflammasome pathways can exert deleterious effects through up-regulation, hyperactivation, and polymorphisms. Polymorphisms and mutations in the NLRP3 gene have been implicated in various cancers, such as invasive colorectal cancer and pancreatic cancer [29]. Our identification of robust NLRP3 inflammasome expression in Erosive OLP patients aligns with prior research [10]. Nevertheless, to our knowledge, there is limited documentation of Gal-3 expression in OLP. Gal-3, induced in response to pro-inflammatory and anti-inflammatory effects during chronic inflammatory diseases, holds promise as a novel marker of inflammation [30]. Intriguingly, Gal-3 expression exhibits variation across different tissues and cells, with its localization—whether in the cytoplasm, nucleus, or extracellular fluid linked to its diverse biological functions. A previous study reported decreased Gal-3 expression in tissue nuclear specimens but increased cytoplasmic expression in patients with tongue carcinoma [31], suggesting that Gal-3 localization may play a pivotal role in cancer progression. In our study of OLP patients, elevated Gal-3 expression was predominantly observed in the cytoplasm of cells in the lamina propria beneath the basement membrane. Previous studies have implicated strong involvement of Gal-3 in the malignant transformation of precancerous lesions. Considering OLP as an OPMD with the potential to progress into OSCC [32], the significantly increased expression of both Gal-3 and the NLRP3 inflammasome may potentially indicate a trend towards the malignant transformation of OLP, given the well-established connection between inflammation and cancer [33].

**Fig. 4:**
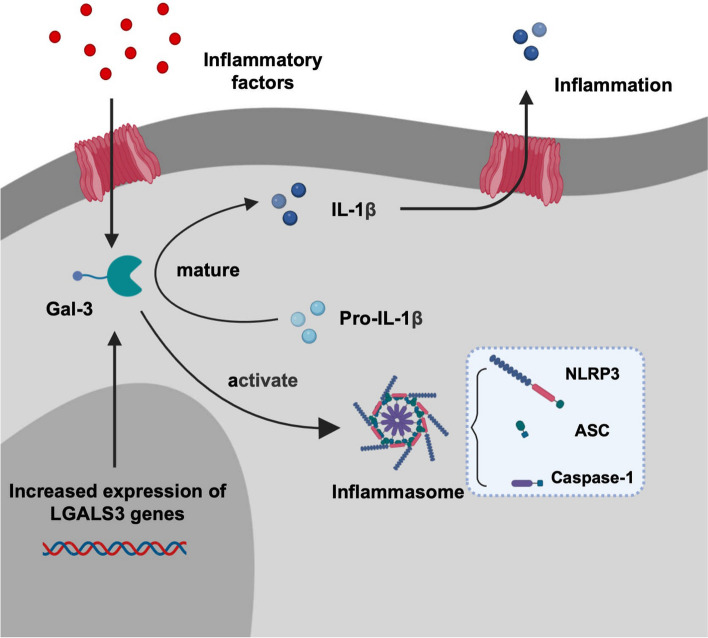
The proposed roles of galectin-3 (Gal-3) in oral lichen planus (OLP). Increased expression of Gal-3 plays an important pro-inflammatory role in the induction phase of OLP by promoting activation of the NLR family pyrin domain containing 3 (NLRP3) inflammasome and the maturation of Interleukin-1β (IL-1β). The figure was created using BioRender.com

In the current investigation, a noteworthy positive correlation was identified between the expression of Gal-3 and NLRP3 in the entire sample (*r* = 0.92, *p* < 0.01). Gal-3 assumes a critical role in modulating innate and adaptive immune functions, concurrently regulating T-cell functions through the inhibition of T-cell apoptosis [34, 35]. Additionally, NLRP3 is implicated in the regulation of helper T cell activation [36]. An examination of livers in patients with Primary Biliary Cholangitis (PBC) revealed Gal-3’s increased expression and NLRP3 activation to be Gal-3 dependent [37]. Gal-3 is known to play a pivotal pro-inflammatory role during the induction phase of acute colitis, facilitating NLRP3 inflammasome activation and subsequent IL-1β production [17]. Researchers have further elucidated that the N-terminal domain of Gal-3 directly binds to NLRP3, thereby activating the inflammasome. Gal-3-induced inflammasome activation results in IL-1β production, subsequently enhancing IL-17 production by macrophages in an autocrine manner [37]. Consequently, Gal-3 and the NLRP3 inflammasome may be implicated in the activation of T lymphocytes in OLP. Building upon the amalgamation of prior research findings and the current investigation, we propose a conceivable mechanism for the onset and progression of OLP (Fig. 4).

Several limitations warrant consideration in this study. Firstly, despite the modest sample size, the study successfully identified significant differences in Gal-3 and NLRP3 expression between groups, along with a robust correlation between Gal-3 and NLRP3. Future investigations will extend the sample size for more comprehensive insights. Secondly, the cross-sectional nature of this study precludes the determination of causal relationships. Thirdly, potential selection bias in the clinic setting is acknowledged. Given that patients with OLP inherently present with comorbidities, and recognizing the differential expression of Gal-3 and NLRP3 in various immune-mediated diseases and inflammatory disorders, patients with alternative immune-mediated chronic inflammatory conditions were intentionally excluded to ensure sample homogeneity in this study.

## Conclusion

The protein expression levels of Gal-3 was increased in OLP lesions and there was a positive correlation between Gal-3 and the NLRP3 inflammasome. Overall, this study provides a histological basis for Gal-3 and NLRP3 inflammasome involvement in the initiation of OLP. Further studies, such as quantitative analysis, are needed to clarify the use of Gal-3 and NLRP3 expression as new markers that may indicate a pro-inflammatory state during chronic inflammatory diseases and cancer transformation.

### Supplementary Information

**Additional file 1: Table S1. **Pearson correlation coefficients among the galectin-3 (Gal-3) and NLR family pyrin domain containing 3 (NLRP3) inflammasome in the whole sample. **Fig. S1. Expression of ASC. **Expression of apoptosis-associated speck-like protein containing a CARD (ASC) in oral lichen planus (OLP) lesions (**A**) and normal oral mucosa (**B**). Scale bars: 100 μm (left); 50 μm (right). The percentage of ASC positive cells in tissues of OLP patients was significantly higher than that in the control group; t-test; *p* < 0.0001 (**C**).** Fig. S2. Expression of Caspase-1. **Expression of Caspase-1 in oral lichen planus (OLP) lesions (**A**) and normal oral mucosa (**B**). Scale bars: 100 μm (left); 50 μm (right). The percentage of Caspase-1 positive cells in tissues of OLP patients was significantly higher than that in the control group; t-test; *p* < 0.0001 (**C**).

## Data Availability

The datasets used and/or analyzed during the current study are available from the corresponding author on reasonable request.

## Abbreviations

OLP: Oral lichen planus
Gal-3: Galectin-3
NLRP3: NLR family pyrin domain-containing 3 inflammasome
OSCC: oral squamous cell carcinoma
ASC: apoptosis-associated speck-like protein containing a CARD
PBC: primary biliary cholangitis
NLR: NOD-like receptor
NOD: Nucleotide-binding oligomerization domain
