# Supplementary material for: Inter-relationships of galectin-3 and NLR family pyrin domain containing 3 inflammasomes with oral lichen planus: a preliminary cross-sectional in vitro study
Source: BMC Oral Health. 2024 Jan 3;24:14. doi: 10.1186/s12903-023-03780-8 (PMC10765663; doi:10.1186/s12903-023-03780-8)
Supplement: Supplementary file 1 — Additional file 1: Table S1. Pearson correlation coefficients among the galectin-3 (Gal-3) and NLR family pyrin domain containing 3 (NLRP3) inflammasome in the whole sample. Fig. S1. Expression of ASC. Expression of apoptosis-associated speck-like protein containing a CARD (ASC) in oral lichen planus (OLP) lesions (A) and normal oral mucosa (B). Scale bars: 100 μm (left); 50 μm (right). The percentage of ASC positive cells in tissues of OLP patients was significantly higher than that in the control group; t-test; p < 0.0001 (C). Fig. S2. Expression of Caspase-1. Expression of Caspase-1 in oral lichen planus (OLP) lesions (A) and normal oral mucosa (B). Scale bars: 100 μm (left); 50 μm (right). The percentage of Caspase-1 positive cells in tissues of OLP patients was significantly higher than that in the control group; t-test; p < 0.0001 (C). [file 12903_2023_3780_MOESM1_ESM.docx]

**Table S1** Pearson correlation coefficients among the galectin-3 (Gal-3) and NLR family pyrin domain containing 3 (NLRP3) inflammasome in the whole sample

|  | NLRP3^1^ | ASC^2^ | Caspase-1 | |
| --- | --- | --- | --- | --- |
|  | Pearson correlation coefficients | | |  |
| Gal-3^3^ | 0.92** | 0.90** | 0.89** |  |

^1^ NLR family pyrin domain containing 3

^2^ Apoptosis-associated speck-like protein containing a CARD

^3^ galectin-3

**Significant correlations were established at *p*-value<0.01


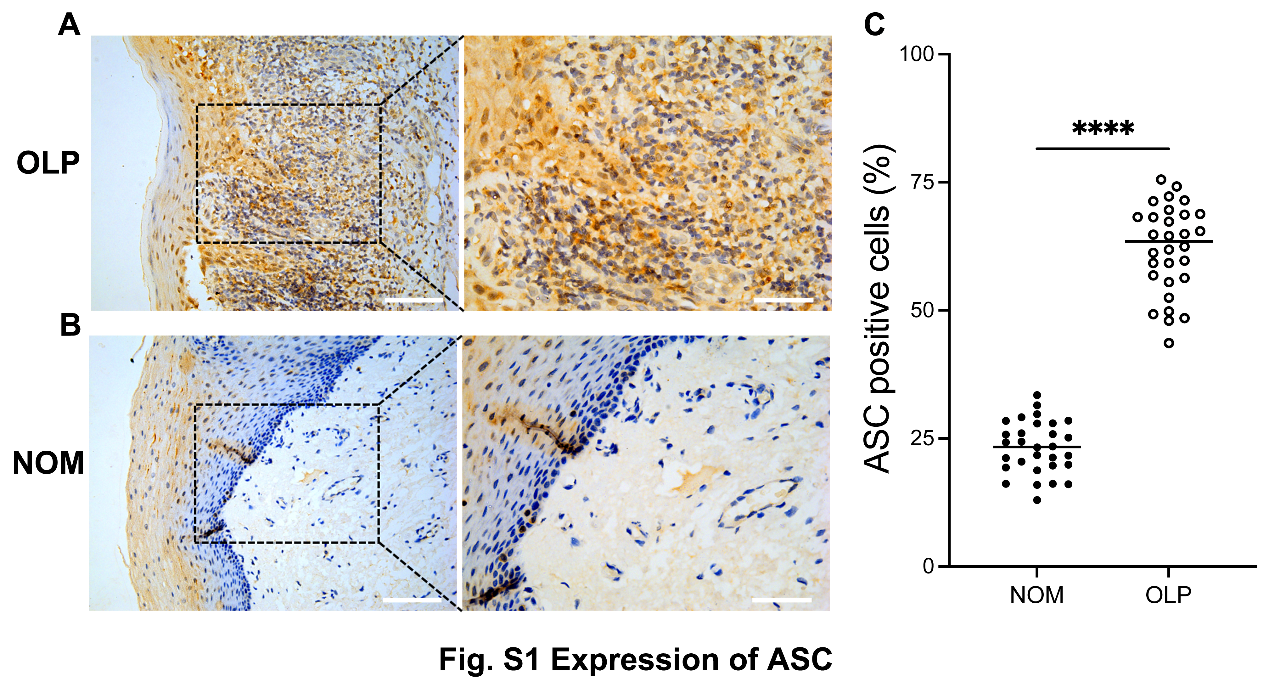
**Fig. S1** Expression of apoptosis-associated speck-like protein containing a CARD (ASC) in oral lichen planus (OLP) lesions (a) and normal oral mucosa (b). Scale bars: 100 μm (left); 50 μm (right). The percentage of ASC positive cells in tissues of OLP patients was significantly higher than that in the control group; t-test; *p* < 0.0001 (c)


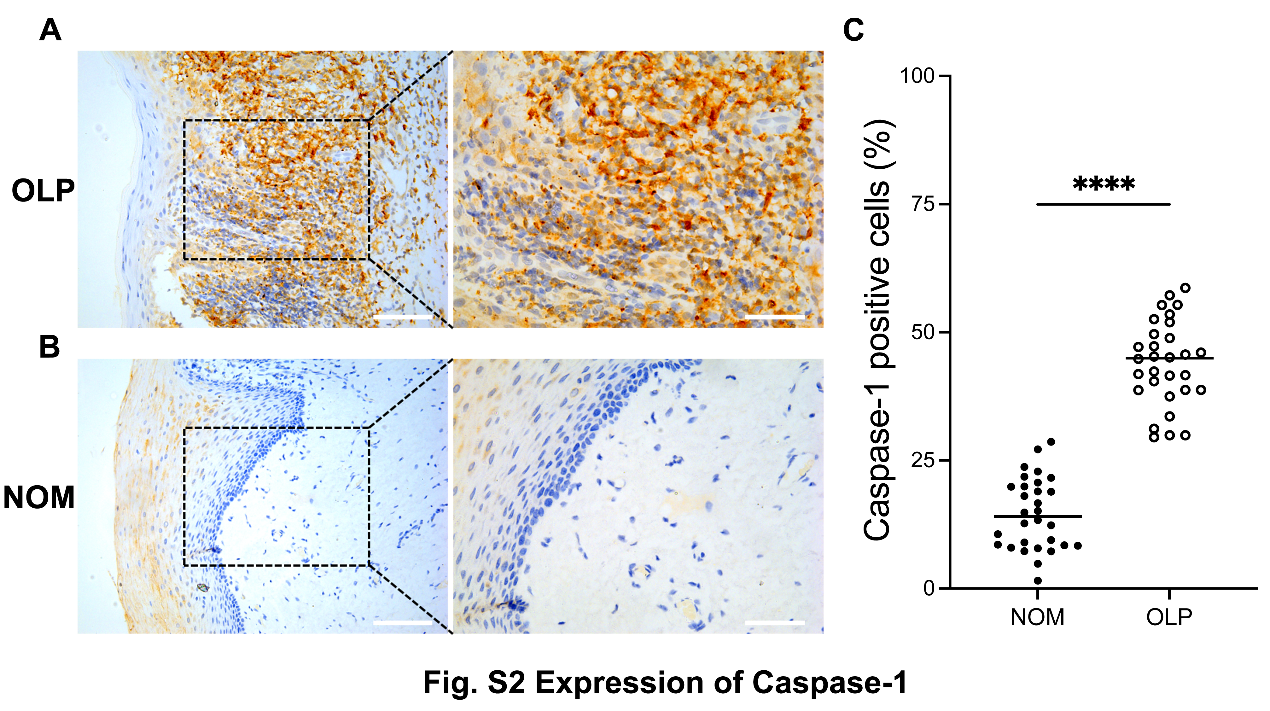
**Fig. S2** Expression of Caspase-1 in oral lichen planus (OLP) lesions (a) and normal oral mucosa (b). Scale bars: 100 μm (left); 50 μm (right). The percentage of Caspase-1 positive cells in tissues of OLP patients was significantly higher than that in the control group; t-test; *p* < 0.0001 (c)
